# Polyvinyl alcohol coating prevents platelet adsorption and improves mechanical property of polycaprolactone-based small-caliber vascular graft

**DOI:** 10.3389/fcvm.2022.946899

**Published:** 2022-08-11

**Authors:** Naohiro Wakabayashi, Takumi Yoshida, Kyohei Oyama, Daisuke Naruse, Masahiro Tsutsui, Yuta Kikuchi, Daisuke Koga, Hiroyuki Kamiya

**Affiliations:** ^1^Department of Cardiac Surgery, Asahikawa Medical University, Asahikawa, Japan; ^2^Life Materials Development Section, Human Life Technology Research Institute, Toyama Industrial Technology Research and Development Center, Toyama, Japan; ^3^Business Development Section, Department of Business Development and Quality Control, Iaazaj Holdings Co., Ltd., Toyama, Japan; ^4^Department of Microscopic Anatomy and Cell Biology, Asahikawa Medical University, Asahikawa, Japan

**Keywords:** small vascular graft, polyvinyl alcohol, platelet adhesion, polycaprolactone (PCL), nanofiber

## Abstract

The low patency of synthetic vascular grafts hinders their practical applicability. Polyvinyl alcohol (PVA) is a non-toxic, highly hydrophilic polymer; thus, we created a PVA-coated polycaprolactone (PCL) nanofiber vascular graft (PVA–PCL graft). In this study, we examine whether PVA could improve the hydrophilicity of PCL grafts and evaluate its *in vivo* performance using a rat aorta implantation model. A PCL graft with an inner diameter of 1 mm is created using electrospinning (control). The PCL nanofibers are coated with PVA, resulting in a PVA–PCL graft. Mechanical property tests demonstrate that the PVA coating significantly increases the stiffness and resilience of the PCL graft. The PVA–PCL surface exhibits a much smaller sessile drop contact angle when compared with that of the control, indicating that the PVA coating has hydrophilic properties. Additionally, the PVA–PCL graft shows significantly less platelet adsorption than the control. The proposed PVA–PCL graft is implanted into the rat’s abdominal aorta, and its *in vivo* performance is tested at 8 weeks. The patency rate is 83.3% (10/12). The histological analysis demonstrates autologous cell engraftment on and inside the scaffold, as well as CD31/α-smooth muscle positive neointima regeneration on the graft lumen. Thus, the PVA–PCL grafts exhibit biocompatibility in the rat model, which suggests that the PVA coating is a promising approach for functionalizing PCL.

## Introduction

Ischemic heart disease has been a major cause of death for decades, causing over 8.9 million deaths globally in 2019 (1). Surgical revascularizations such as coronary artery bypass grafting (CABG) are the first line of treatment in severe cases of this disease, and autologous graft is the only clinically approved approach (2). However, autologous graft has various limitations, such as invasion on harvest, limited availability of grafts of the required length, as well as availability issues in patients whose vessels have already been harvested. Therefore, the development of a synthetic vascular graft with a small diameter (less than 5 mm) has been in great demand in cardiovascular surgery (2).

An ideal synthetic vascular graft integrates with the host tissue and behaves similarly to a native vascular vessel in terms of self-regeneration and ability to grow. To this end, the use of biocompatible nanofibers has been gaining attention in vascular tissue engineering (VTE), as it can provide a scaffold for the host, allowing cell engrafting and autologous regeneration (2, 3). Polycaprolactone (PCL) is a synthetic biodegradable polymer and the most widely used in VTE because of its mechanical properties (2). However, the hydrophobic characteristic of PCL causes protein and platelet adhesion and can lead to acute thrombotic obstruction, especially in small-diameter grafts (4, 5). Therefore, researchers have been exploring methods to provide antiplatelet functions, for example, conjugation of an antiplatelet reagent (heparin) to inhibit platelet aggregation, bonding factors, or chemokines for recruiting endothelial progenitors, and blending with other natural polymers to add biocompatibility (6–11). However, providing sufficient hemocompatibility to PCL grafts remains an open issue.

Polyvinyl alcohol (PVA) contains a hydroxyl group in its structure and is a highly hydrophilic polymer, unlike many other synthetic polymers (12). Because of its proven non-toxic and biodegradable characteristics, PVA has been applied in biomedical areas, for example, drug delivery carriers, soft contact lenses, artificial joints, and cell therapy (13, 14). In addition, many biomedical applications are being developed, including wound dressings, tissue adhesion barriers, and artificial kidneys (15–17), by using the unique feature of PVA, namely, low protein adsorption property (18). Recently, it has been proposed that the presence of intermediate water on a biocontact interface is crucial for preventing non-specific adsorption of biocomponents, and hydrophilic polymers can form such a water layer to increase hemocompatibility (19, 20), which can explain the mechanism of PVA showing its high biocompatibility. In vascular research, heparin is the most studied molecule for adding hemocompatibility. A major pharmacological property of heparin is binding and activating antithrombin to accelerate the inhibition of thrombin and factor Xa with its negatively charged sulfonic and carboxylic groups. Although PVA does not possess negatively charged functional groups, given the characteristics of PVA described above, we hypothesized that PVA can add hemocompatibity to a vascular graft with a mechanism different from heparin.

Therefore, we developed a PCL nanofiber-based vascular graft functionalized with PVA. In this study, we aimed to (1) clarify whether the PVA coating can prevent platelet adhesion on PCL grafts and (2) characterize its patency and autologous tissue-like regeneration in a rat implantation model.

## Materials and methods

### Polycaprolactone graft preparation and polyvinyl alcohol coating

A PCL nanofiber sheet was fabricated by electrospinning a solution of 10% PCL (Mw 80,000, Sigma-Aldrich) dissolved in *N, N*-dimethylformamide and tetrahydrofuran (3:7 w:w) in accordance with our previous study (6). The spinning conditions were as follows: 20 kV voltage, 20 cm tip collector distance, 1.7 mL/h flow rate, and 20 rpm spinning rate. The PCL sheet was cut into 20 mm widths and wound on a 1 mm axle of polytetrafluoroethylene (PTFE) to form an inner layer. The second rectangular sheet was wound on the initial layer in the opposite direction (middle layer), and the outer layer was molded in the same direction as the inner layer. The PTFE axle was removed, and the resulting tubular scaffold was the PCL graft.

PVA (10 g) (606021; Kaneyo-Soap Co., Ltd., Japan) was dissolved in water (10 g) at room temperature. Subsequently, 10 g of ethanol was added to the solution. One milliliter of the solution was sucked up with a 5 ml syringe connected to a tapered nozzle. The taper nozzle was inserted into the PCL graft, and the solution was slowly extruded. This procedure was repeated twice. Air was then blown using an empty syringe to remove the excess solution remaining in the graft. Finally, the grafts were dried overnight at room temperature (approximately 25°C).

### Observation of detailed structure of graft

The grafts were cut using single-edge industrial blades (T586; EMJapan Co., Ltd., Japan). The cut samples were mounted on aluminum bases, coated with gold using an ion-sputter coater (JEC-550; JEOL Ltd., Japan), and observed under a scanning electron microscope (JSM-6610LA; JEOL Ltd., Japan).

### Mechanical property measurement

The mechanical properties of the PCL graft were measured using a Kawabata evaluation system (KES) (21) (Supplementary Figures 1A,B) with a compression tester (KES-F3-A, Kato Tech Co., Ltd., Japan). The sample was compressed at a constant velocity (0.02 mm/s) until the compressive force reached 50 gf/cm^2^. Then, the compressor was set back to release the force at the same velocity. The stress–strain curve (Supplementary Figure 1B) was obtained by plotting the compressed thickness and compressive load of the samples on the *x*- and *y*-axis, respectively. Upon compression, the curve extends to A (the point corresponding to the maximal compressive force: 50 gf/cm^2^) from O (the point corresponding to the absence of compressive force). The curve then descends according to the removal of the compressive force and finally reaches B, which is the point corresponding to the complete removal of the compressive force. When the sample completely recovers its form, point B corresponds to point O. C represents the compressed thickness at the maximal compressive force. The area under the curve (AUC) of the upper line obtained under compression is denoted by “a,” and “b” denotes the AUC of the lower line obtained from releasing the compressive force. From these values, the compression work (WC), compression resiliency (RC), and T_0_–T_M_ were obtained using the following formula: WC = a, RC = b/a, T_0_–T_M_ = Length [O-C]. WC is an indicator of the compression susceptibility. A higher value indicates that the sample is more susceptible to compression and collapses easily. RC and T_0_–T_M_ can be used to evaluate the resiliency or recoverability; the sample is observed to have enhanced elastic properties as the values of RC and T_0_–T_M_ approach 100 and 0, respectively.

### Hydrophilicity test

The hydrophilicity of the PCL surface was evaluated using sessile drop contact angle measurement (22). A drop of deionized water (3.1 μl) was placed on the PCL sheets coated with or without PVA. The contact angle (*θ*) was measured using the half-angle method (23). Briefly, the angle (*θ*1) from the edge to the vertex of the drop on the contact surface was assumed to be half of the geometrical contact angle (*q* = 2*θ*1) because a small and light drop behaves as a sphere. From the values of the radius (r) and height (h) of the sphere measured using a contact angle meter (CA-X, Kyowa Interface Science Co., Ltd., Japan), the contact angle was calculated as tan *θ*1 = h/r, *θ* = 2 arctan [h/r] (Supplementary Figure 1C). The contact angle represents the wettability (hydrophilicity) of the material surface; a smaller contact angle indicates greater hydrophilicity.

### Platelet adhesion

This study was approved by the institutional review board of Asahikawa Medical University (approval number: 21166 on 3/18/2022). Written informed consent was obtained from all participants.

Blood harvested from healthy volunteers was centrifuged at 200 × g for 5 min at 4°C to obtain the platelet-rich plasma. Two milliliters of the plasma were run into a PCL graft (1 cm length, 1 mm inner diameter) three times, and the graft was flushed with 10 ml of phosphate-buffered saline (PBS, pH 7.4) to wash out the platelet-rich plasma. The inside of the graft was observed using an electron microscope to evaluate the platelet adhesion. The grafts were placed in 2% glutaraldehyde solution (0.1 M phosphate buffer (PB), pH 7.4) at 4°C for 24 h. For conductive staining, they were immersed in 1% tannic acid (0.1 M PB) for 1 h, rinsed with a buffer solution for 1 h, and immersed in 1% osmium tetroxide (0.1 M PB) for 1 h. The specimens were subsequently dehydrated using a graded ethanol series (70, 80, 90, and 95%, 30 min each). After dehydration, t-butyl alcohol was substituted with ethanol (10 mm × three times) and dried in a freeze dryer (ID-2; EIKO Co., Japan). The dried specimens were mounted onto aluminum bases, coated with platinum-palladium using an ion-sputter coater (E1030; Hitachi Koki Co., Japan), and observed with a field-emission scanning electron microscope (S-4100; Hitachi High-Technologies, Japan).

### Graft implementation and harvest

All animal experiments were approved by the Institutional Animal Care and Use Committee of Asahikawa Medical University (reference number 20129-2).

Male Wistar rats (8–9 weeks old, 280–320 g) were purchased from the Charles River animal facility (Japan) and anesthetized with 2.0% isoflurane. The abdominal aorta was isolated through a midline laparotomy. After clamping the aorta below the renal arteries on the proximal side and above the aortoiliac bifurcation on the distal side, the aorta was transected and replaced with a PCL or PVA–PCL graft with an inner diameter of 1 mm and length of approximately 5–7 mm. Patency at implantation was judged by restoration of the graft form and lower-limb discoloration upon pinching the graft. After closing the abdominal cavity and skin, the rats were kept in individual cages and monitored for 8 weeks. The rats were provided normal food and water, and no anticoagulation or antiplatelet drugs were administered throughout this study. Finally, the grafts were harvested under general anesthesia. The patency of the graft was determined by pulsating the blood flow from a hemi-resection site in the distal aorta of the graft anastomosis. The rats were then euthanized, and the grafts were harvested after injecting 4% paraformaldehyde (PFA) *via* the cardiac apex.

### Histological analysis

The harvested grafts were fixed in 4% PFA overnight at 4°C. The grafts were then cut (i) axially into three sections: proximal, middle, and distal or (ii) longitudinally from the proximal to a distal anastomosis. They were then embedded in an optimal cutting temperature compound (Sakura Finetek Japan Co., Japan). The frozen grafts were sliced with a cryostat to 5 μm thickness at −20°C and used for hematoxylin and eosin (HE) staining and immunofluorescence staining (IF). For IF, heat-induced epitope retrieval was performed using tris-ethylenediaminetetraacetic acid (EDTA) buffer (10 mM Tris base, 1 mM EDTA, 0.05% Tween 20, pH 9.0), and the specimens were blocked with 1% bovine serum albumin in PBS before the occurrence of antibody reactions. Hoechst 33342 (FUJIFILM, 346-07951, Japan) was used for nuclear staining, and the following antibodies were used for IF in this study [1st antibodies: anti-CD31 (R&D SYSTEMS, AF3628, United States, dilution 1:200) and anti-alpha smooth muscle actin (αSMA) (Cell Signaling Technology, 56856, dilution 1:250), 2nd antibodies: Alexa Fluor 488 conjugated anti-goat IgG (Thermo Fisher Scientific, A-11055, United States) and Alexa Fluor 555 conjugated anti-mouse IgG (Thermo Fisher Scientific, A-31570, United States)]. The HE and IF images were captured using an all-in-one fluorescence microscope (Keyence BZ-X810, Japan).

The neointimal thickness was measured with Fiji (24). By using the HE images, the cell layer on the lumen corresponding to the CD31(+)/αSMA(+) layer in the IF images was defined as the neointimal layer. The proximal and distal sites were defined as the regions between 100 and 300 μm from each anastomosis site. The middle site was defined at approximately 3 mm away from the proximal anastomosis site. For each site of each sample, the average thickness of 3 randomly selected parts of the neointima was used as the thickness.

### Statistical analysis

All statistical analyses were performed using GraphPad Prism9. The data were expressed as mean ± standard deviation (SD). Unpaired *t*-tests were used to compare the mean values between the two groups. One-way analysis of variance followed by a *post-hoc* test was performed to compare more than two groups. The statistical significance was set at *p* < 0.05.

## Results

### Fabrication of polyvinyl alcohol-coated polycaprolactone graft

**FIGURE 1:**
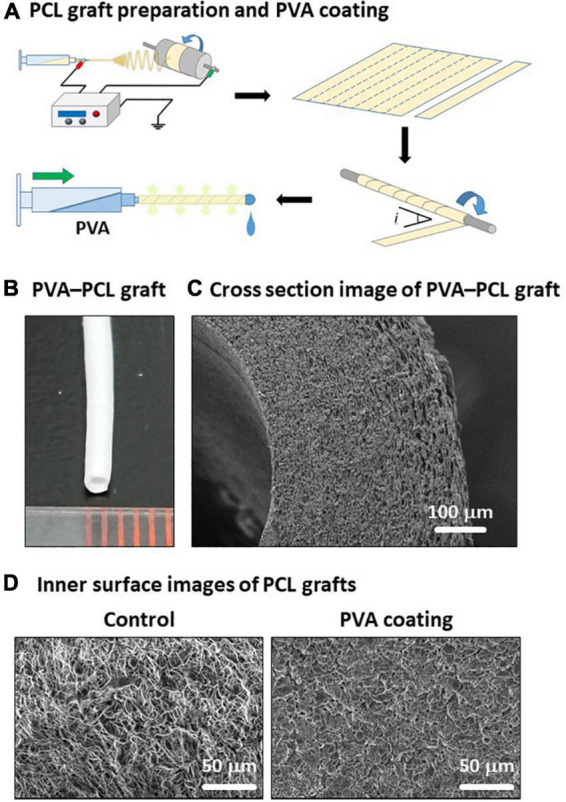
Fabrication of PVA–PCL graft with 1 mm inner diameter. **(A)** Scheme of PCL graft preparation and PVA coating. **(B)** PVA–PCL graft. **(C)** Cross-section of PVA–PCL graft by SEM imaging. **(D)** Comparison of the luminal surface structure before and after PVA coating.

To overcome two issues: (i) technical difficulty in creating a tubular structure with uniform thickness and small diameter, and (ii) hydrophilicity of PCL, we fabricated PVA-coated PCL nanofiber grafts with 1 mm inner diameter as described in Figure 1A (details in section “Materials and methods”). The resulting PVA–PCL graft is depicted in Figure 1B. Cross-sectional imaging of the PVA–PCL graft using scanning electron microscopy (SEM) showed a dense nanofiber scaffold having certain porosity (Figure 1C). The PCL graft without PVA coating (control) showed a rough surface of the inner lumen, while the PVA–PCL graft showed a smoother inner lumen (Figure 1D).

### Polyvinyl alcohol coating increases stiffness and elasticity

**FIGURE 2:**
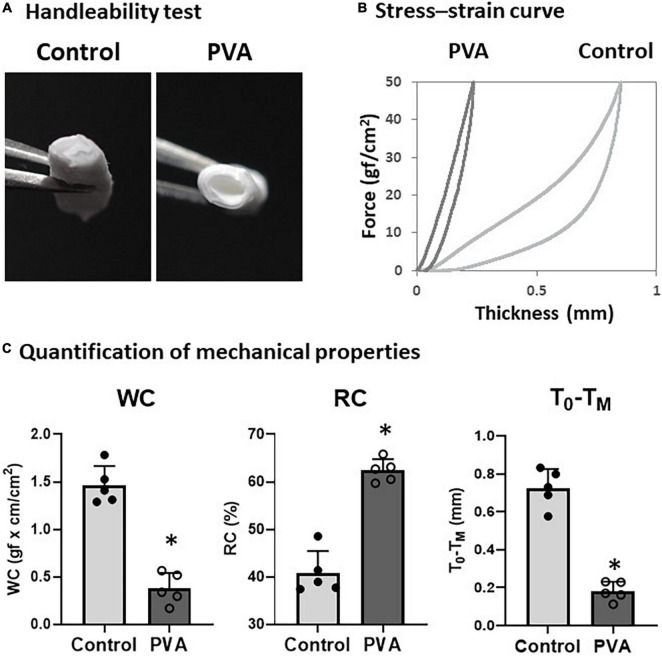
Evaluation of mechanical properties of PVA–PCL graft. **(A)** Handleability test. Grafts were pinched with forceps 5 times and the resulting change in structure was captured. **(B)** Stress–strain curve measured using KES. Grafts were compressed with a maximum force of 50 gf/cm^2^ and the distance between compression and recovery was measured using a compression tester. Independent measurements were repeated 5 times and the figure shows the representative data. **(C)** Quantification of mechanical properties measured by KES. WC represents susceptibility against compression. Higher value of RC and smaller T_0_–T_M_ represents resiliency and recoverability. The graphs are expressed as the mean ± SD and the data points represent the individual values in each group. ^∗^Denotes *p* < 0.05.

We observed a change in the firmness of the PCL graft after the PVA coating. Therefore, we tested the handleability of the PVA–PCL graft using surgical forceps. After repeated pinches with the forceps a few times, the control PCL graft collapsed, and the inner cavity was closed easily (Figure 2A). By contrast, the PVA–PCL graft resisted collapse and retained the inner cavity. Next, we quantified the effect of the PVA coating on the mechanical properties of the PCL graft. The stiffness, resiliency, and recoverability of the PCL graft were measured using the KES method (21) (Figures 2B,C). As shown in Figure 2B, the control PCL graft was compressed to approximately 0.8 mm under the maximum compressive force and gradually recovered upon releasing the force. However, the stress–strain curve of the PVA–PCL graft showed a leftward shift, and the length of compression was approximately 0.3 mm under the maximum force. WC indicates compression susceptibility. The WC values were 0.38 ± 0.15 and 1.46 ± 0.18 for the PVA–PCL and control grafts, respectively; thus, the value was significantly reduced in the PVA–PCL graft. RC represents the recoverability (maximum value is 100%); it was significantly increased in the PVA–PCL graft (62.4 ± 2.4% vs. 40.8 ± 4.6% in the PCL graft). A smaller value of T_0_–T_M_ indicates the stiffness (minimum value is 0 mm); it was significantly decreased in the PVA–PCL graft (0.18 ± 0.05 mm vs. 0.73 ± 0.10 mm in the PCL graft). Thus, consistent with the results of the handleability test, the KES method demonstrated that the PVA coating improved the resilience and mechanical strength of the PCL graft.

### Polyvinyl alcohol coating increases hydrophilicity and prevents platelet adsorption

**FIGURE 3:**
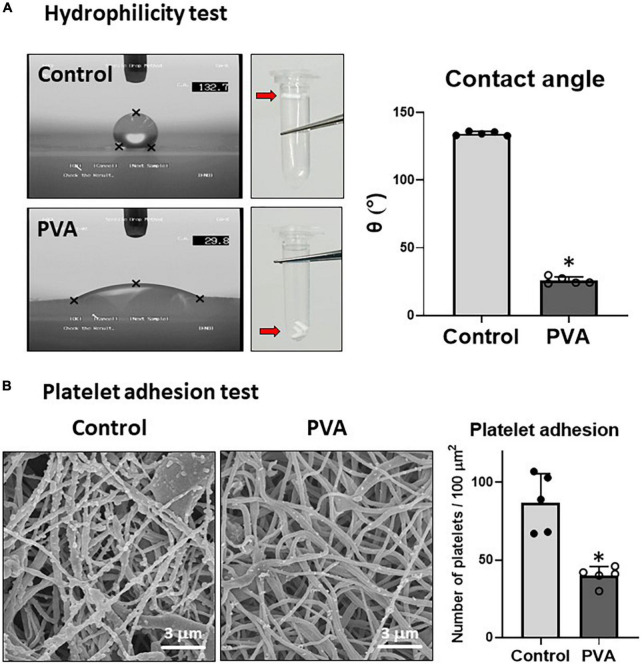
Hydrophilicity and platelet adhesion tests. **(A)** Hydrophilicity test. The hydrophilicity of the control and PVA–PCL surfaces was measured through the sessile drop contact angle method. The sessile drop was imaged using an angle meter. Independent measurements were repeated five times, and the representative images from each group are shown on the left. Three grafts were soaked in PBS, and the results are shown at the center of the figure. The contact angle results are shown on the right. The graphs are expressed as mean ± SD, and the data points represent the individual values in each group. ^∗^Represents *p* < 0.05. **(B)** Platelet adhesion test. Human plasma was passed through the grafts. After flushing with PBS, the adsorbed platelets were imaged using SEM. The independent tests were repeated five times, and the representative images are shown on the left. Numerical results are shown on the right. The graphs are expressed as the mean ± SD, and the data points represent the individual values in each group. ^∗^Represents *p* < 0.05.

PVA is a highly hydrophilic polymer (25). We examined the effect of the PVA coating on the hydrophilicity of the PCL graft by using sessile drop contact angle measurement (8). A drop of water on the control PCL sheet assumed a spherical shape (Figure 3A, upper left). By contrast, the water drop on the PVA-coated PCL sheet had flattened (Figure 3A, lower left). The contact angle decreased significantly in the PVA-coated PCL sheet compared with that of the control (25.9° ± 2.3 vs. 134.3° ± 1.3 in the control). Consistent with the results from the contact angle measurement, the PVA–PCL graft sank quickly upon soaking in water, but the control PCL graft remained floating. Thus, the PVA coating significantly promoted the hydrophilicity and wettability of the PCL graft.

Because hydrophobic surfaces adsorb platelets (5), we tested whether the increased hydrophilicity due to the PVA coating prevented platelet adhesion on the PLC graft. Human platelet-rich plasma was prepared and allowed to flow through the grafts. The adsorbed platelet granules were observed through SEM imaging. The results showed that significantly fewer platelet granules adhered to the inner lumen of the PVA–PCL graft (40 ± 6 platelet granules/100 μm^2^) when compared with that in the control PCL graft (87 ± 17 platelet granules/100 μm^2^) (Figure 3B). Thus, the PVA coating prevented platelet adhesion on the PCL graft.

### Patency and endothelialization of polyvinyl alcohol–polycaprolactone graft *in vivo*

**FIGURE 4:**
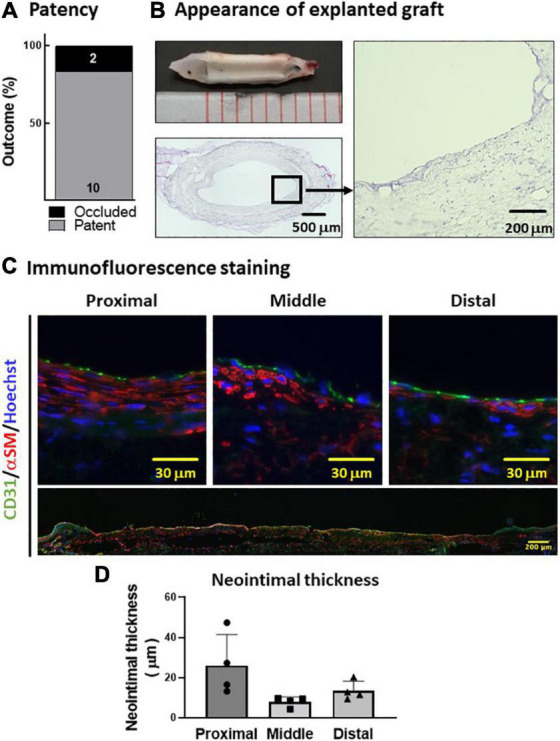
*In vivo* evaluation of PVA–PCL graft performance. **(A)** Patency rate. PVA–PCL grafts were implanted into rat (*n* = 12). Patency was determined after 8 weeks. **(B)** Appearance of explanted graft. Macroscopic appearance (upper left). Cross-sectional HE images: low magnification (lower left) and 400X (right). **(C)** Neointima images after immunofluorescence staining. Endothelial cell marker CD31 (green) and smooth muscle cell marker αSM (red) were used for immunostaining. The nucleus was counter-stained with Hoechst (blue). All harvested grafts were immunostained, and representative data are shown here. Higher magnification images of a cross-sectional graft are shown at the top, and an end-to-end graft image is shown at the bottom with low magnification. **(D)** Quantification of the neointimal thickness measured using cross-sectional HE images. The data are expressed as mean ± SD, and the data points represent the individual values in each group.

Next, we tested the *in vivo* performance of the PVA–PCL graft as a small-diameter vascular graft. The PVA–PCL grafts were implanted into the rat’s abdominal aorta with interposition. The patency rate at 8 weeks was 83.3% (10 of 12) (Figure 4A). The harvested PVA–PCL grafts showed a smooth texture and no thrombus in the lumen in all patent cases (Figure 4B). HE staining was performed to evaluate the recellularization of the PVA–PCL graft (Figure 4C). Cellular engraftment was observed on the lumen, outer surface, inside the PVA–PCL scaffold, and cell layers were formed on the luminal side. Immunofluorescence staining was performed to examine whether the cell layer was neointima. CD31(+)/αSMA(+) cell layers were observed at the proximal, middle, and distal regions of the PVA–PCL graft, suggesting that neointima was regenerated and almost fully covered the graft end-to-end. The neointimal layer tended to be thicker at the anastomosis sites when compared with the middle, and the averaged thicknesses were 26.1 ± 13.3 μm, 8 ± 2.1 μm, and 13.5 ± 4.1 μm at the proximal, middle, and distal sites, respectively. These data demonstrated that the PVA–PCL graft retained patency beyond 8 weeks with autologous tissue-like regeneration, including the neointimal structure.

## Discussion

We developed a PVA–PCL graft, where PCL nanofibers were coated with hydrophilic polymer PVA and demonstrated that (1) the PVA coating increased the mechanical properties and hydrophilicity of the PCL graft and (2) the PVA–PCL graft exhibited patency beyond 8 weeks with neointima regeneration.

### Polyvinyl alcohol coating decreased platelet adhesion

We demonstrated that the PVA coating added hydrophilicity to the PCL graft and significantly reduced the platelet adsorption (Figure 3B). It has been well documented that small-diameter vascular grafts fail quickly because of thrombus adherence and vascular obstruction (9, 22). Platelet activation is the initial step of the thrombosis cascade and is, therefore, a significant cause of thrombotic occlusion of vascular grafts. To overcome this issue and improve the hemocompatibility of blood-contacting materials, many research groups have employed a chemical modification of the graft surfaces using materials such as heparin (7, 8, 26) and polyethylene glycol (PEG) (10, 27). A common feature of these modifications is hydrophilicity. Heparin is a glycosaminoglycan that exhibits high hydrophilicity in addition to its pharmacological effect of activating antithrombin (28). PEG is a hydrophilic polyether, and Deible et al. demonstrated that the tethering of PEG onto various material surfaces, including glass, polyethylene, and PTFE, significantly prevented platelet deposition (27). As demonstrated in these reports, hydrophilicity is a key requirement for biocompatibility and prevents the non-specific adsorption of biocomponents, including platelets and serum proteins (29). As a mechanism, it has been proposed recently that the presence of freezing bound water on the surface of artificial material plays an important role in preventing non-specific binding of biological components (19, 20). Considering these findings, the hydrophilic nature of PVA is likely a mechanism where PVA coating prevents platelet adhesion on a PCL graft. Our results suggested that employing PVA is a promising approach to achieve hemocompatibility to polymer-based vascular grafts including PCL.

### Improved mechanical property due to polyvinyl alcohol coating

Mechanical property tests indicated that the PVA–PCL graft was stiffer and more elastic than the control PCL graft. In the PVA–PCL graft, the voids between the PCL nanofibers are filled with PVA, and the PCL nanofibers are bonded together, which increases the graft density and limits the deviation occurring between the fibers during deformation. This is presumably the reason the PVA–PCL graft demonstrates improved mechanical properties and requires greater force to deform.

As a vascular graft, mechanical properties such as stiffness and elasticity are of paramount importance because the graft is anastomosed to the native vessels with a needle suture. In our preliminary study, the PCL graft without PVA coating was used for the replacement of the abdominal aorta in the same rat model as applied in the present study. However, the PCL graft was fragile, and anastomosis was extremely difficult because the graft was easily broken during the process, resulting in very poor early patency (approximately 30%), even though these experiments were performed by cardiac surgeons with adequate experience. Although there is no standardized method and it is difficult to quantify the physical experience of the anastomosis operation, from the viewpoint of a cardiac surgeon, the mechanical properties of the PVA–PCL graft used in this study appear to be sufficient and satisfactory for anastomosis.

### Mechanism of neointima formation

Nanofiber-based scaffolds are believed to be ideal for VTE because they can provide biomimetic microenvironments for cells to regenerate tissue (7, 8, 11, 20). Consistent with this concept, the PVA–PCL graft showed cell engraftment inside the scaffold and CD31(+)/αSMA(+) neointima formation (Figure 4). At 8 weeks after implantation, the PVA–PCL graft lumen was almost fully covered by neointima; however, the neointima tended to be thinner in the middle of the graft when compared with the regions surrounding the anastomosis sites, suggesting that the migration of the intima layer from a native vessel is a potential mechanism of neointima formation, which is also supported by another study (30). Neointima formation is particularly important for long-term patency, and this result is encouraging as a vascular graft. Although ePTEF graft is a clinically established vascular graft for middle to large size revascularization, it has limited autologous cell engraftment. It is interesting to speculate whether applying PVA-PCL nanofiber to the lumen of ePTFE vascular graft could allow endothelialization. Future study will be needed to prove this concept.

### Limitations of this study

Although this study demonstrated that PVA improved the functional and mechanical properties of PCL graft, and PVA-PCL graft displayed biocompatibility with neointima regeneration, some limitations remained. (1) In this study, PVA solution was infused into the PCL graft for coating. Since PVA was not immobilized, we speculate that PVA disappears at some point due to its solubility and degradability, and we did not clarify the duration for which the effect of PVA is maintained *in vivo*. In our future study, further investigations on how to sustain the function for longer periods will be undertaken. In this regard, making blended polymer is one of the options. However, to our knowledge, it is difficult to make blended polymer of PVA and PCL because solvents for PVA and PCL are aqueous and organic, respectively, and do not mix. In contrast, there is a report on fabricating copolymer of PVA and PCL (31). In this paper, the biocompatibility was tested by mouse fibroblast 3T3 culture, however, hydrophilicity, and anti-platelet function were not determined. It will be interesting to investigate if copolymers of PVA and PCL have the same effect, as our study demonstrated; if they do, this strategy could be widely applicable for clinical usage. (2) The PVA–PCL graft implanted in this study was 5–7 mm in length, however, the required length for clinical application is much longer. For example, graft length of 10–15 cm is needed for CABG, and would presumably require a longer time to regenerate neointima end-to-end. This could be a critical factor in losing patency and must be addressed. Thus, further studies to characterize the time course of neointima formation and long-term patency of PVA–PCL grafts by using larger animal models are needed.

## Conclusion

We developed a PCL graft functionalized with a hydrophilic PVA polymer and tested its graft performance in a rat aorta implantation model. The PVA coating added hydrophilic properties to the PCL graft, which suppressed platelet adsorption. An *in vivo* performance test showed that the PVA–PCL graft with an inner diameter of 1 mm was patent beyond 8 weeks and demonstrated autologous tissue regeneration, including the neointima layer. Our data suggest that functionalization of the PCL grafts with PVA is a promising approach for increasing hemocompatibility. Considering the development of a clinical application, further animal model tests are needed to understand and overcome the limiting factors of graft failure, such as graft length, long-term patency, performance under disease conditions, and manner of anastomosis.

## Data availability statement

The original contributions presented in this study are included in the article/Supplementary material, further inquiries can be directed to the corresponding author/s.

## Ethics statement

The animal study was reviewed and approved by the Institutional Animal Care and Use Committee of Asahikawa Medical University.

## Author contributions

NW and TY performed data collection, analysis, and manuscript writing. MT and YK helped NW with data collection. KO performed data analysis, manuscript writing, and coordination of the study. TY and DN performed material preparation. DK performed scanning electron microscope imaging. HK conceived the study and reviewed the manuscript. All authors read and approved the final manuscript.
