# Supplementary material for: Polyvinyl alcohol coating prevents platelet adsorption and improves mechanical property of polycaprolactone-based small-caliber vascular graft
Source: Front Cardiovasc Med. 2022 Aug 11;9:946899. doi: 10.3389/fcvm.2022.946899 (PMC9403249; doi:10.3389/fcvm.2022.946899)
Supplement: Supplementary file 1 [file Image_1.pdf]

Supplementary Material

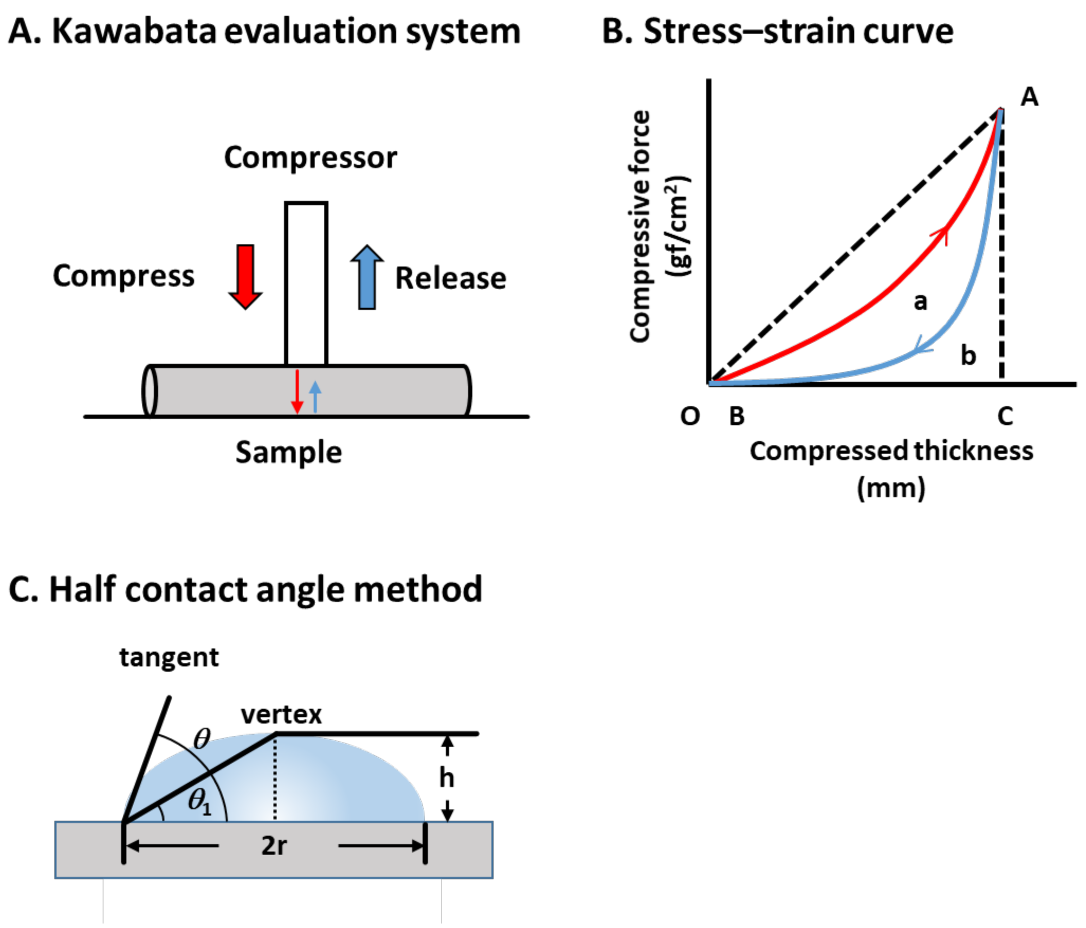

Supplementary Figure 1. Methods to evaluate mechanical property and hydrophilicity (A) Graphical image of KES method. Compressor compresses the specimen with a constant velocity until the compressive force reaches the maximum, and it moves back at constant velocity to release the force. The compressed and recovered distance of the specimen is continuously monitored to make the stress-strain curve. (B) Stress-strain curve. The red line indicates the compressed distance from O to A. Blue line indicates the recovered distance from A to B. C indicates the compressed distance with maximum force (50 gf/cm^2). a = area under the curve (AUC) of the red line obtained from compression, b = AUC of the blue line obtained from release of the compressive force. Mechanical property parameters are calculated as follows: WC = a, RC = b/a, T0-TM = Length [O-C]. (C) Graphical description of half contact angle method. Theoretical contact angle (θ) is calculated as follows: $\tan \theta_1 = h/r$, $\theta = 2 \arctan [h/r]$.
